# Determinants of the persistency of macrosomia and shoulder dystocia despite treatment of gestational diabetes mellitus

**DOI:** 10.1016/j.heliyon.2020.e03756

**Published:** 2020-04-09

**Authors:** Cécile Pénager, Pascal Bardet, José Timsit, Jacques Lepercq

**Affiliations:** a Department of Obstetrics, Cochin-Port-Royal Hospital, AP-HP, Paris Descartes University, DHU Risks in Pregnancy, 123 boulevard de Port-Royal, 75014, Paris, France; b Department of Diabetology, Cochin-Port-Royal Hospital, AP-HP, Paris Descartes University, DHU Authors, 123 boulevard de Port-Royal, 75014, Paris, France

**Keywords:** Health sciences, Women's health, Obstetrics, Pregnancy, Reproductive system, Gestational diabetes mellitus, Macrosomia, Blood glucose self-monitoring, Blood glucose control

## Abstract

**Aims:**

to identify potentially modifiable risk factors associated with the persistency of macrosomia and/or shoulder dystocia in infants born to women treated for gestational diabetes mellitus (GDM).

**Methods:**

this case-control retrospective study included 113 cases complicated by macrosomia (ponderal index ≥97^th^ percentile) and/or shoulder dystocia, and 226 controls without these complications. Factors associated with macrosomia and/or shoulder dystocia and with failure of diabetes management were assessed by multivariable analyses.

**Results:**

Macrosomia and/or shoulder dystocia were associated with previous delivery of a large for gestational age (LGA) infant (adjusted odds ratio, 2.34, 95% confidence interval [1.01–5.45]), three abnormal glucose values during oral glucose tolerance test (2.83 [1.19–6.72]), a higher gestational weight gain before treatment (1.08 [1.01–1.15]), and failure of diabetes management (2.68 [1.32–5.45]). A non-Euro Caucasian origin (3.08 [1.37–6.93]), previous delivery of a LGA infant (3.21 [1.31–7.87]), institution of treatment after 32 weeks of gestation (3.92 [1.86–8.25]), and insulin therapy (2.91 [1.20–7.03]) were associated with failure of diabetes management.

**Conclusions:**

supportive care in at risk women, limitation of weight gain in early pregnancy, shortened delay between diagnosis and treatment of GDM, and intensive insulin dosage adjustments might be means to improve the neonatal prognosis of GDM.

## 1 Introduction

Gestational diabetes mellitus (GDM) is associated with an increased risk of adverse neonatal outcomes, mainly macrosomia, shoulder dystocia and birth trauma [1]. The role of maternal hyperglycemia in the occurrence of these complications has been demonstrated by intervention studies aiming at normalizing maternal blood glucose values [2, 3]. A recent meta-analysis confirmed that, compared with routine care, treatment of GDM significantly reduces the incidence of macrosomia and of shoulder dystocia [4]. Despite treatment of GDM, increased rates of adverse neonatal outcomes persist. In a recent population-based study, the risks of macrosomia (odds ratio, 1.6) and of birth injury (odds ratio 1.2) were increased compared to pregnancies not complicated by GDM [5]. This may be due to risk factors other than blood glucose control, as demonstrated for an increased prepregnancy body weight or an excessive gestational weight gain [6, 7, 8]. However, few studies assessed the impact of the quality of maternal blood glucose control on the occurrence of these adverse outcomes [9, 10].

The aim of our study was to identify risk factors associated with macrosomia and/or shoulder dystocia in infants born to women treated for GDM, with special emphasis on potentially modifiable factors such as observance of self-monitoring of capillary blood glucose (SMBG) and achievement of blood glucose targets.

## 2 Material and methods

### 2.1 Study design

This retrospective case-control study was performed in women with GDM who gave birth between January 1, 2013 and December 31, 2014 in the department of obstetrics, Cochin-Port-Royal hospital.

According to French recommendations, selective screening for GDM was performed in women with at least one of the following risk factors: age ≥35 years, body mass index (BMI) ≥ 25 kg/m^2^, family history of diabetes in at least one first-degree relative, personal history of GDM, previous delivery of a large for gestational age (LGA) infant [11]. Screening for GDM was performed as follows: fasting plasma glucose was measured at the first prenatal visit and an oral glucose tolerance test (OGTT) with 75 g of glucose with glycemic measurements at 0, 1 and 2 h was performed between 24-28 weeks of gestation (WG) if previous fasting glucose was normal or had not been performed. GDM was defined according to the International Association of the Diabetes and Pregnancy Study Groups criteria as regards early fasting plasma glucose and glucose values at OGTT [12].

All women with GDM participated in an outpatient teaching program including information about GDM, the expected benefits of treatment and its modalities, and education to diet management and SMBG. Women were asked to perform daily SMBG at least fasting and 2 h after the three main meals. Blood glucose targets were <5.3 mmol/l before meals and <6.7 mmol/l 2-h postprandial. When blood glucose targets were not achieved after 7–10 days, insulin therapy was initiated. Basal and/or prandial insulin injections were instituted according to individual glycemic profiles and women were taught to adjust doses every two days to reach blood glucose targets. Data were collected from standardized medical files by two of us (C.P., J.L.).

### 2.2 Subjects

**Figure 1:**
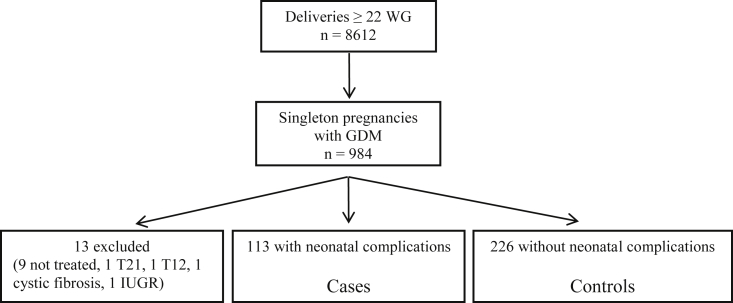
Study profile. WG, weeks of gestation; GDM, gestational diabetes mellitus; T21, trisomy 21; T12, trisomy 12; IUGR, intrauterine growth restriction. Among the 8612 women who delivered during the study period, GDM was diagnosed in 984 (11.4%). Thirteen women were excluded from the study: nine because GDM was not treated, one who had cystic fibrosis, two who delivered infants with a chromosomal anomaly, and one case of severe fetal growth restriction. Macrosomia (n = 102) and/or shoulder dystocia (n = 16) occurred in 113 of the 971 infants (11.6%). Controls were women with GDM who gave birth to children without these complications. Each case was matched with the two controls who delivered next to it, at the same gestational age ±1 WG.

Women with GDM and a single pregnancy who delivered a live-birth infant after 22 WG were eligible for the study (Figure 1). Women with pre-existing diabetes and those who underwent a termination of pregnancy for severe congenital malformation, or experienced stillbirth, were excluded from the study. The National Data Protection Authority (Commission Nationale de l’Informatique et des Libertés, CNIL n° 1755849) approved this study. Under French regulations, this study was exempt from institutional ethics review because it is an observational study using anonymized data from medical records. Women were informed that their records can be used for the evaluation of medical practices and were explicitly informed that they can opt out of these studies.

Two groups were defined. Cases were women who delivered a macrosomic infant, defined by a ponderal index (PI), calculated as birth weight in g x 100/(height in cm)^3^, ≥97^th^ percentile, i.e., 3 g/cm^3^ [13]; and/or the occurrence of shoulder dystocia, defined as failure to deliver the fetal shoulder(s) with gentle downward traction on the fetal head requiring additional obstetric maneuvers to effect delivery, as reported in the medical files; and/or the occurrence of birth injury (clavicle fracture, brachial plexus injury). Controls were women with GDM who gave birth to children without these complications. For each case we included two controls who delivered next to it, at the same gestational age ±1 WG (Figure 1).

The following characteristics were recorded: age; prepregnancy body weight and BMI; ethnicity; employment; smoking status; history of diabetes in first-degree relatives; parity; GDM and/or delivery of a LGA infant in a previous pregnancy; gestational weight gain; gestational age and blood glucose values at diagnosis of GDM; gestational age at initiation of GDM treatment, and at insulin therapy; pre- and postprandial SMBG values and daily insulin dosage averaged over 7 days between 32 and 38 WG; gestational age and route of delivery; birth weight and height; percentile of birth weight [14]; occurrence of shoulder dystocia or birth injury; neonatal hypoglycemia, defined by a capillary blood glucose ≤2.6 mmol/l at 3 h of life [15]; respiratory distress syndrome; hyperbilirubinemia requiring phototherapy.

Observance of SMBG and the quality of blood glucose control were assessed over 7 days between 32 and 38 WG. Poor observance of SMBG was defined as less than 21 measures performed of the at least 28 required. Poor glucose control was defined as a mean fasting blood glucose level ≥5.3 mmol/l and/or mean 2-h postprandial blood glucose level ≥6.7 mmol/l, or when specifically indicated in the patient's file by the referring consultant. We defined "failure of GDM management" as a poor observance of SMBG and/or a poor glucose control.

### 2.3 Statistical analysis

Data were expressed as medians and interquartile ranges (IQR), or as numbers and percentages. Univariable analyses were performed using the Mann-Whitney non-parametric test, and the Fisher's exact test for categorical variables. Associations between the exposure variables and the occurrence of outcomes (i.e., macrosomia and/or shoulder dystocia and/or birth injury) were assessed by multivariable logistic regression. Risk factors described in the literature, and the variables of our analysis that seemed clinically and statistically relevant, were entered in the model. Exposure variables included: a Euro Caucasian origin, previous delivery of a LGA infant, pregestational BMI, the number of abnormal glucose values at OGTT, gestational age at treatment, weight gain before treatment, insulin therapy and failure of GDM management. Collinearity was tested using the variance inflation factor (VIF). Crude and adjusted ORs were calculated with their 95% confidence intervals (CI). Since the ponderal index is not universally used to define macrosomia, we also performed a sensitivity analysis using birth weight >90^th^ percentile to define macrosomia. Statistical analyses were performed with STATA 11.0 (*Stata Statistical Software: Release 11*. College Station, TX: StataCorp LP).

## 3 Results

### 3.1 Main characteristics of the study population

Among the 8612 women who delivered during the study period, GDM was diagnosed in 984 (11.4%). Thirteen women were excluded from the study: nine because GDM was not treated, one who had cystic fibrosis, two who delivered infants with a chromosomal anomaly, and one case of severe fetal growth restriction (Figure 1).

Macrosomia (n = 102) and/or shoulder dystocia (n = 16) occurred in 113 of the 971 infants (11.6%). Two birth injuries were associated with shoulder dystocia. Among the 339 women included in the study, 153 (45%) were of Euro Caucasian origin, 108 (32%) had a family history of diabetes, and 176 (52%) were multiparous. Among the multiparous women, 45 (26%) had a history of GDM, 47 (27%) had previously given birth to a LGA infant, and 65 (37%) had been delivered by cesarean section. At the time of the studied pregnancy, median age of the women was 35 years (IQR 31–39), pregestational body weight was 68 kg (IQR 58–80), and BMI was 25 kg/m^2^ (IQR 21–29).

### 3.2 Comparisons of women and GDM characteristics according to the occurrence of macrosomia and/or shoulder dystocia

In the univariable analysis, there were more women of non-Euro Caucasian origin, more multiparous women and more women who previously delivered a LGA infant among the cases than in the controls (Table 1). The rate of GDM diagnosed during the first trimester of pregnancy was similar in the two groups (14% vs. 13%, *P* = 0.73). Fasting blood glucose and the number of abnormal values at OGTT were higher among the cases. Weight gain before institution of treatment was higher in the cases than in the controls. Treatment of GDM (diet and SMBG) was instituted slightly later in the cases. Insulin therapy was more frequent in the cases, with no significant differences in gestational age at its institution or in insulin doses. Poor observance of SMBG and poor glucose control were three times more frequent in the cases than in the controls.

**Table 1:** Main characteristics of the mothers of infants with (cases) or without (controls) macrosomia and/or shoulder dystocia: univariable analysis. Results are medians and interquartile ranges into brackets with actual numbers into parentheses, or actual numbers with percentages into parentheses.

	Cases (113)	Controls (226)	*P*
Age (years)	35 [30–38] (113)	35 [31–39] (226)	0.3242
Euro Caucasian origin: no/yes	73/40 (65%)	112/113 (50%)	0.0109
Family history of diabetes: yes/no	38/75 (34%)	70/156 (31%)	0.6231
Employment: no/yes	28/85 (25%)	43/183 (19%)	0.2574
Smoking: yes/no	5/108 (4.4%)	18/208 (8%)	0.2595
Multiparous: yes/no	69/44 (61%)	107/119 (47%)	0.0210
Personal history of GDM: yes/no	21/48 (30%)	24/82 (23%)	0.2896
Previous delivery of a LGA infant: yes/no	29/40 (42%)	18/89 (17%)	0.0004
Previous cesarean delivery: yes/no	27/84 (24%)	38/184 (17%)	0.1422
Pregestational BMI (kg/m^2^)	25 [22.8–29.3] (112)	24 [21–29] (225)	0.1634
Pregestational BMI: normal/overweight/obese	49/35/28	114/64/47	0.4705
Fasting glucose value at 1^st^ trimester (mmol/l)	5.6 [4.9–5.9] (22)	5.6 [5.4–5.8] (29)	0.8120
Glucose values at OGTT (mmol/l)			
0′	5.2 [4.7–5.4] (97)	4.9 [4.6–5.1] (195)	0.0281
60′	9.9 [8.8–10.7] (90)	9.7 [8.5–10.5] (187)	0.3177
120′	8.7 [7.9–9.4] (92)	8.6 [7.4–9.3] (192)	0.1664
Number of abnormal glucose values at OGTT			
1	44 (49%)	118 (63%)	
2	26 (29%)	55 (29%)	0.0016
3	20 (22%)	14 (8%)	
Weight gain before treatment of GDM (kg)	9 [4–13] (109)	7 [3–10] (218)	0.0202
Gestational age at treatment (WG)	28 [27–34] (113)	28 [25–32] (223)	0.0363
Insulin therapy: yes/no	26/81 (24%)	31/194 (14%)	0.0201
Gestational age at insulin therapy (WG)	30 [25–33] (25)	30 [26–32] (30)	0.9528
Insulin doses∗: UI/kg.d.	0.38 [0.14–0.59] (22)	0.45 [0.33–0.58] (24)	0.2963
Weight gain after treatment (kg)	2 [1–4] (97)	2 [0–4] (198)	0.9247
Total weight gain (kg)	12 [7.5–15] (103)	10 [6–14] (203)	0.0399
Observance of SMBG: no/yes	15/94 (14%)	9/217 (4%)	0.0024
Achievement of good glucose control: no/yes	32/74 (30%)	21/201 (9%)	<10^−4^
Failure of GDM management†: yes/no	41/72 (36.3%)	28/198 (12.4%)	<10^−4^

GDM, gestational diabetes mellitus; LGA, large for gestational age; BMI, body mass index; OGTT, oral glucose tolerance test; WG, weeks of gestation; SMBG, self-monitoring of blood glucose.

∗ average values over one week between 32 and 38 WG.

† defined as a poor observance of SMBG and/or a poor glucose control.

In the multivariable analysis, previous delivery of a LGA infant (aOR, 2.34, 95%CI [1.01–5.45]), three abnormal glucose values during OGTT (aOR, 2.83, 95%CI [1.19–6.72]), a higher weight gain before treatment of GDM (aOR, 1.08, 95%CI [1.01–1.15]), and failure of GDM management (aOR, 2.68, 95%CI [1.32–5.45]) remained independently associated with the occurrence of macrosomia and/or shoulder dystocia (Table 2). The VIFs of exposure variables kept in the model were between 1.1 and 1.5, excluding multicollinearity; they were between 5.4 and 5.8 for multiparity, fasting glycemia at OGTT, and total weight gain, which were removed from the model.

**Table 2:** Factors associated with the occurrence of macrosomia and/or shoulder dystocia in infants born to mothers with GDM: multivariable analysis.

	crude ORs [95% CI]	adjusted OR [95% CI]	*P*
Euro Caucasian origin: no vs yes	1.84 [1.16–2.93]	1.08 [0.60–1.95]	0.788
Previous delivery of a non-LGA infant vs no previous delivery	1.22 [0.73–2.02]	0.77 [0.41–1.44]	0.413
Previous delivery of LGA vs non-LGA infant	3.59 [1.79–7.20]	2.34 [1.01–5.45]	0.049
Pregestational BMI	1.02 [0.98–1.06]	1.04 [0.98–1.09]	0.216
Number of abnormal glucose values at OGTT			
1	REF	-	
2	1.27 [0.71–2.27]	1.13 [0.60–2.13]	0.704
3	3.83 [1.78–8.24]	2.83 [1.19–6.72]	0.019
Gestational age at treatment	1.04 [0.99–1.08]	1.03 [0.96–1.11]	0.394
Weight gain before treatment	1.05 [1.02–1.10]	1.08 [1.01–1.15]	0.017
Insulin therapy: yes vs no	2.01 [1.12–3.60]	1.09 [0.46–2.63]	0.840
Failure of GDM management∗: yes vs no	4.03 [2.32–6.99]	2.68 [1.32–5.45]	0.007

LGA, large for gestational age; BMI, body mass index; OGTT, oral glucose tolerance test; GDM, gestational diabetes mellitus.

∗ defined as a poor observance of self-monitoring of blood glucose and/or a poor glucose control.

In the sensitivity analysis using a birth weight >90^th^ percentile instead of a ponderal index ≥97^th^ percentile, the same variables remained associated with the occurrence of macrosomia and/or shoulder dystocia (Table 3).

**Table 3:** Sensitivity analysis of the factors associated with a birth weight >90^th^ percentile and/or the occurrence of shoulder dystocia.

	Cases (65)	Controls (274)	*P*
Age (years)	34 [30–38] (65)	35 [31–39] (274)	0.2837
Euro Caucasian origin: no/yes	43/22 (66%)	142/131 (52%)	0.0517
Family history of diabetes: yes/no	24/41 (37%)	84/190 (31%)	0.3746
Employment: no/yes	17/48 (26%)	54/220 (20%)	0.3084
Smoking: yes/no	2/63 (3%)	21/253 (8%)	0.2731
Multiparous: yes/no	42/23 (65%)	134/140 (49%)	0.0270
Personal history of GDM: yes/no	12/29 (29%)	33/101 (25%)	0.5464
Previous delivery of a LGA infant: yes/no	22/20 (52%)	25/109 (19%)	<10^−4^
Previous caesarean delivery: yes/no	19/45 (30%)	46/223 (17%)	0.0339
Pregestational BMI (kg/m^2^)	25 [23–31] (65)	24 [21–29] (272)	0.0689
Pregestational BMI: normal/overweight/obese	26/20/19	137/79/56	0.2254
Fasting BG value at 1^st^ trimester (mmol/l)	5.7 [5.4–6.0] (13)	5.6 [5.3–5.8] (38)	0.4115
BG values at OGTT (mmol/l)			
0′	5.2 [4.6–5.4] (55)	5.0 [4.6–5.4] (237)	0.1905
60′	9.5 [8.8–10.9] (50)	9.8 [8.5–10.5] (227)	0.3538
120′	8.8 [7.7–9.7] (51)	8.7 [7.4–9.3] (233)	0.1348
Number of abnormal BG values at OGTT			
1	25 (50%)	137 (60%)	
2	13 (26%)	68 (30%)	0.0202
3	12 (24%)	22 (10%)	
Weight gain before treatment (kg)	10 [4.4–13] (64)	7 [3–10] (263)	0.0027
Gestational age at treatment (WG)	29.5 [26–34] (65)	28 [25.5–32] (271)	0.0838
Insulin therapy: yes/no	15/46 (25%)	42/229 (15%)	0.0937
Gestational age at insulin therapy (WG)	30 [23–33] (14)	30 [26–32] (41)	0.6566
Insulin doses∗: UI/kg.d.	0.46 [0.13–0.60] (13)	0.42 [0.30–0.58] (33)	0.6430
Weight gain after treatment (kg)	2 [1–4.3] (56)	2 [0–4] (239)	0.9910
Total weight gain	13 [7–17] (60)	10 [7–13] (246)	0.0071
Observance of CBG-SM: no/yes	11/54 (17%)	17/257 (6%)	0.0099
Achievement of good BG control: no/yes	21/40 (34%)	32/235 (12%)	<10^−4^
Failure of GDM management†: yes/no	25/40 (38%)	44/230 (16%)	0.0001

Results are medians and interquartile ranges into brackets with actual numbers into parentheses, or actual numbers with percentages into parentheses.

GDM, gestational diabetes mellitus; LGA, large for gestational age; BMI, body mass index; BG, blood glucose; OGTT, oral glucose tolerance test; WG, weeks of gestation; CBG-SM, capillary blood glucose self-monitoring.

∗ average values over one week between 32 and 38 WG.

† defined as a poor observance of CBG-SM and/or a poor glucose control.

### 3.3 Other adverse perinatal outcomes

As regards other perinatal outcomes, there were more cesarean deliveries, mainly planned, in the cases than in the controls. Respiratory distress syndrome was more frequent among the cases, but there were no differences in the frequency of neonatal hypoglycemia and in the need for phototherapy (Table 4).

**Table 4:** Neonatal outcomes in children with (cases) or without (controls) macrosomia and/or shoulder dystocia.

	Cases (113)	Controls (226)	*P*
Gestational age at delivery (WG)	39 [38–40] (113)	39 [38.3–40] (226)	0.3281
Caesarean delivery: yes/no	54/59 (48%)	65/160 (29%)	0.0007
Caesarean delivery: planned/emergency	36/18 (67%)	29/35 (45%)	0.0260
Birth trauma: yes/no	2/111	0/226	-
CBG value at 3 h of life (mmol/l)	3.2 [2.9–3.4] (93)	3.3 [2.9–3.7) (182)	0.0852
Neonatal hypoglycemia: yes/no	15/78 (16%)	21/161 (12%)	0.3448
Need for phototherapy: yes/no	6/106 (5%)	4/221 (1.7%)	0.0889
Respiratory distress syndrome: yes/no	10/102 (9%)	4/221 (1.7%)	0.0033

Results are medians and interquartile ranges into brackets with actual numbers into parentheses, or actual numbers with percentages into parentheses.

WG, weeks of gestation; CBG, capillary blood glucose.

### 3.4 Determinants of the failure of GDM management

Since failure of GDM management was a major factor associated with macrosomia and/or shoulder dystocia, we compared the characteristics of the women who had a poor observance of SMBG and/or a poor glucose control to that of the others. In the univariable analysis, a non-Euro Caucasian origin, absence of employment, multiparity, previous delivery of a LGA infant, pregestational BMI, fasting blood glucose and the number of abnormal values at OGTT, institution of treatment after 32 WG, and insulin therapy were all associated with failure of GDM management (Table 5). In the multivariable analysis, a non-Euro Caucasian origin (aOR, 3.08, 95%CI [1.37–6.93]), previous delivery of a LGA infant (aOR, 3.21, 95%CI [1.31–7.87]), institution of treatment after 32 WG (aOR, 3.92, 95%CI [1.86–8.25]), and insulin therapy (aOR, 2.91, 95%CI [1.20–7.03]) were all associated with failure of GDM management (Table 6). A poor glucose control was more frequent in insulin-treated women than in those on diet (38% vs. 12%, OR 4.39, 95% CI 2.28–8.45, *P* < 10^−4^).

**Table 5:** Factors associated with the failure of GDM management: univariable analysis.

	Failure of GDM management	No failure of GDM management	*P*
n	69	270	
Age (years)	35 [31–39] (69)	35 [31–39] (270)	0.7076
Euro Caucasian origin: no/yes	58/11 (84%)	127/142 (47%)	<10^−4^
Family history of diabetes: yes/no	24/45 (35%)	84/186 (31%)	0.5651
Employment: no/yes	24/45 (35%)	47/223 (17%)	0.0026
Smoking: yes/no	4/65 (6%)	19/251 (7%)	1.0000
Multiparous: yes/no	45/24 (65%)	131/139 (49%)	0.0151
Personal History of GDM: yes/no	16/29 (36%)	29/101 (22%)	0.1122
Previous delivery of a LGA infant: yes/no	21/24 (47%)	26/105 (20%)	0.0008
Pregestational BMI (kg/m^2^)	26 [24–31] (68)	24 [21–28] (269)	0.0012
Pregestational BMI: normal/overweight/obese	23/22/23	140/77/52	0.0105
Fasting glucose at 1^st^ trimester (mmo/l)	5.7 [5.4–6.3] (14)	5.6 [5.3–5.8] (37)	0.1511
glucose values at OGTT (mmol/l)			
0′	5.3 [4.9–5.6] (57)	4.9 [4.5–5.3] (228)	<10^−4^
60′	9.5 [8.8–10.7] (53)	9.7 [8.5–10.5] (226)	0.5488
120′	8.7 [7.3–9.6] (55)	8.7 [7.6–9.3] (231)	0.7147
Number of abnormal glucose values at OGTT			
1	27 (53%)	135 (60%)	0.0053
2	11 (22%)	70 (31%)	
3	13 (25%)	21 (9%)	
Gestational age at treatment (WG)	28 [26–34.5] (67)	28 [26–32] (269)	0.3513
Treatment after 32 WG: yes/no	25/42 (37%)	56/213 (21%)	0.0066
Weight gain before treatment (kg)	9 [3.3–13] (62)	8 [4–11] (265)	0.3214
Insulin therapy: yes/no	23/45 (34%)	34/230 (13%)	0.0001
Gestational age at insulin therapy (WG)	30 [23.5–33] (22)	30 [26–33] (33)	0.8635
Insulin doses∗: UI/kg.d.	0.57 [0.36–0.59] (18)	0.37 [0.17–0.57] (28)	0.1733

Results are medians and interquartile ranges into brackets with actual numbers into parentheses, or actual numbers with percentages into parentheses.

GDM, gestational diabetes mellitus; LGA, large for gestational age; BMI, body mass index; OGTT, oral glucose tolerance test; WG, weeks of gestation.

∗ average values over one week between 32 and 38 WG.

**Table 6:** Factors associated with failure of GDM management: multivariable analysis.

	crude ORs [95% CI]	adjusted ORs [95% CI]	*P*
Euro Caucasian origin: no vs yes	5.89 [2.96–11.73]	3.08 [1.37–6.93]	0.007
Employment: no vs yes	2.53 [1.41–4.55]	1.30 [0.61–2.77]	0.490
Previous delivery of a non-LGA infant vs no previous delivery	1.32 [0.71–2.46]	1.02 [0.48–2.21)	0.951
Previous delivery of LGA vs non-LGA infant	4.68 [2.28–9.11]	3.21 [1.31–7.87]	0.011
Pregestational BMI (kg/m^2^)	1.06 [1.02–1.11]	1.05 [0.99–1.11]	0.083
Number of abnormal glucose values at OGTT			
2 vs 1	0.79 [0.37–1.68]	0.62 [0.27–1.40]	0.247
3 vs 1	3.10 [1.38–6.93]	1.66 [0.68–4.03]	0.267
Gestational age at treatment >32 WG	2.26 [1.27–4.03]	3.92 [1.86–8.25]	<10^−4^
Insulin therapy: yes vs no	3.46 [1.86–6.42]	2.91 [1.20–7.03]	0.018

LGA, large for gestational age; BMI, body mass index; OGTT, oral glucose tolerance test; WG, weeks of gestation.

## 4 Discussion

In our study, we identified several factors associated with macrosomia and/or shoulder dystocia, namely previous delivery of a LGA infant, three abnormal values at OGTT, weight gain before institution of GDM treatment, and failure of diabetes management. A non-Euro Caucasian origin, late institution of GDM treatment and the need for insulin therapy were associated with failure of diabetes management.

Two baseline characteristics of the women, namely previous delivery of a LGA infant and three abnormal glucose values at OGTT were associated with macrosomia and/or shoulder dystocia. Prior delivery of a LGA infant is a known risk factor for recurrence, both in women without and with GDM [10, 16, 17]. Three abnormal glucose values at OGTT, that could reflect more severe forms of GDM, were also associated with a higher prevalence of these complications. This observation is in keeping with the results of several studies showing worse neonatal outcomes in women with three abnormal glucose values at OGTT [8, 18].

We identified two potentially modifiable risk factors, i.e., gestational weight gain before institution of treatment, and failure of GDM management. Gestational weight gain is a known determinant of neonatal complications [19], and studies have suggested that reducing weight gain may decrease the rate of macrosomia in GDM [3, 20, 21]. However, in these studies the influence of weight gain was assessed after the diagnosis of GDM. Whether an earlier intervention might decrease the rate of neonatal complications deserves further studies. Failure of GDM management was a strong risk factor for macrosomia and/or shoulder dystocia, since poor observance of SMBG and non-achievement of recommended blood glucose targets were both strongly associated with these complications. In a retrospective case-control study, the frequency of neonatal complications and of macrosomia were higher in 1188 women with sub-optimal blood glucose control, compared to 2030 women with well-controlled GDM [9]. In the Metformin for Gestational Diabetes Trial, higher postprandial blood glucose values were independently associated with the delivery of a macrosomic infant [10].

In our study, a non-Euro Caucasian origin, previous delivery of a LGA, late institution of treatment, and insulin therapy were associated with failure of diabetes management.

In a recent study, women with GDM of non-French ethnicity had poorer observance of SMBG and inadequate timing of postprandial measurements compared to Euro Caucasian women [22]. However, this study did not demonstrate any impact of poor observance on pregnancy outcomes, except for a higher rate of late preeclampsia [22]. This may be due to the universal GDM screening performed in that study, which may have identified less severe forms of GDM than in our study.

As regards previous delivery of a LGA infant, there is limited data about its potential impact on the maternal behavior during further pregnancies. A qualitative study showed that a macrosomic infant was perceived as healthy by some women [23], which may generate barriers to adequate GDM management. This is strengthened by the observation that most women with history of macrosomia did not change their behavior as regards weight gain in the following pregnancy [24].

Two potentially modifiable determinants of GDM management failure were also identified, namely late institution of treatment, particularly after 32 WG, and insulin therapy. It has been suggested that good glycemic control should be obtained before 32 WG to prevent macrosomia [25, 26]. In our study, treatment was instituted after 32 WG in 37% of the women with failure of GDM management, suggesting that efforts should be made to shorten this delay. A potential role for health care policies was ruled out by the full coverage of healthcare for all pregnant women in France. As regards insulin therapy, three observational studies suggested that GDM treatment with insulin, compared to nutritional therapy alone, may be associated with a poorer prognosis, including a higher rate of macrosomia. In those studies, blood glucose values upon treatment were not reported [5, 27, 28], although 3^rd^ trimester HbA1c was slightly higher (by 3 mmol/mol, 0.2%) in women treated with insulin in one study [28]. In our study, insulin therapy, gestational age at its institution, and insulin doses were not independently associated with an increased risk of macrosomia and/or shoulder dystocia. However, insulin therapy was independently associated with GDM management failure. More specifically, failure to reach blood glucose targets was observed in 38% of insulin-treated women. Of note, insulin doses were similar in women with and without achieved blood glucose targets, underlying insufficient insulin dosage in the latter. This could be due to fear of hypoglycemia, or insufficient skills to adjust insulin dosage at a time of pregnancy when insulin needs may increase rapidly. These observations suggest that close evaluation of SMBG results, support, and renewed education are needed when insulin therapy is instituted in women with GDM. In this context, the value of telemedicine for improving GDM prognosis remained to be determined [29].

Our study has several limitations. Its single-Centre setting could minimize extrapolation of our results. Also, due to its retrospective design there were missing values. Macrosomia was defined using the ponderal index, which is not universally used. The ponderal index has been shown to delineate symmetric and asymmetric macrosomia better than birth weight [30]. Symmetric macrosomic infants have a normal PI and metabolic parameters similar to that of infants with a birth weight appropriate for their gestational age [18]. By contrast, infants with a PI ≥ 97^th^ percentile have asymmetric macrosomia associated with a higher fat mass, and cord blood insulin and leptin levels [31]. Indeed, as compared to symmetric macrosomia, asymmetric macrosomia has been associated with an increased risk of neonatal complications in infants born to women with diabetes in several studies [32, 33, 34]. Furthermore, in the Hyperglycemia and Adverse Pregnancy Outcomes study, 78% of the infants defined as macrosomic by the LGA criterion were born to women without GDM, according to IADPSG criteria [35].

## 5 Conclusion

Our results suggest that supportive care in non-Euro Caucasian women, limitation of gestational weight gain in early pregnancy, shortened delay between diagnosis and treatment of GDM, and intensive insulin dosage adjustments might be means to decrease the rate of macrosomia and/or shoulder dystocia in GDM.

## Declarations

### Author contribution statement

C. Penager: Conceived and designed the experiments; Analyzed and interpreted the data; Wrote the paper.

J. Lepercq and J. Timsit: Conceived and designed the experiments; Performed the experiments; Analyzed and interpreted the data; Wrote the paper.

P. Bardet: Performed the experiments; Analyzed and interpreted the data.

### Funding statement

This research did not receive any specific grant from funding agencies in the public, commercial, or not-for-profit sectors.

### Competing interest statement

The authors declare no conflict of interest.

### Additional information

TBC No additional information is available for this paper.
